# Belimumab in the Treatment of Connective Tissue Disease-Associated Interstitial Lung Disease: Case Report and Literature Review

**DOI:** 10.7759/cureus.19218

**Published:** 2021-11-02

**Authors:** John Mwangi, Chase Litteken, Ramya Gorthi, Yeswanth Attoti, Rama Atluri

**Affiliations:** 1 Pulmonary and Critical Care Medicine, Saint Louis University School of Medicine, Saint Louis, USA; 2 Internal Medicine, Saint Louis University School of Medicine, Saint Louis, USA; 3 Internal Medicine, St Luke's Hospital, Saint Louis, USA; 4 Rheumatology, Saint Louis University School of Medicine, Saint Louis, USA

**Keywords:** interstitial lung disease, belimumab, pulmonary hypertension, ground-glass opacities, nonspecific interstitial pneumonitis, systemic lupus erythromatosus, connective tissue disease associated interstitial lung disease, scleroderma

## Abstract

Interstitial lung disease or ILD can be described as inflammation, fibrosis, or scarring of the lung’s interstitial, resulting in dyspnea. ILD represents a group of heterogeneous parenchymal lung disorders with complex pathophysiology, differentiated by the clinical and radiological patterns. ILD is one of the most serious pulmonary complications associated with connective tissue diseases (CTDs), resulting in significant morbidity and mortality. Nonspecific interstitial pneumonia is the most common morphological and pathological pattern of ILD seen in CTDs. There are limitations in the therapeutic options resulting in significant morbidity. Certain biologic therapies are being evaluated for the various forms of ILD. The ILD, in this case, is associated with systemic lupus erythematosus (SLE) and scleroderma overlap that was effectively treated with belimumab, a recombinant monoclonal antibody against the B-cell activating factor (B-lymphocyte stimulator).

## Introduction

Interstitial lung disease (ILD) is one of the most serious pulmonary complications associated with connective tissue diseases (CTDs), resulting in significant morbidity and mortality [1]. Nonspecific interstitial pneumonia (NSIP) is one of the morphological and pathological forms seen in association with CTD, hypersensitivity pneumonitis, and can be drug induced or idiopathic. Histologically, the NSIP pattern is characterized by a uniform distribution of interstitial inflammation and fibrosis. The disease presents with varying levels of chronic interstitial inflammation with varying levels of interstitial fibrosis. CTD-associated ILD (CTD-ILD) has no effective treatment, as current therapeutic regimens only slow down disease progression, thus leaving patients with considerable functional disability. Different agents including biological therapies are being investigated in patients with different forms of ILD. Here we discuss a case of CTD-ILD treated with belimumab, a recombinant monoclonal antibody directed against B-cell activating factor.

This article was previously presented as an abstract at the 2020 ATS Annual Scientific Meeting.

## Case presentation

**Figure 1:**
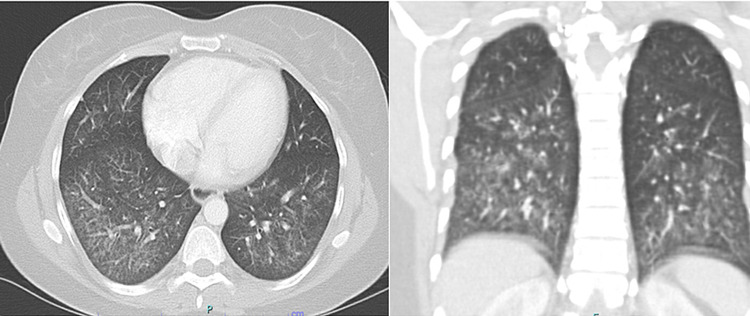
Initial high-resolution CT scan of the chest showing bilateral lower lobes predominant ground-glass opacity (coronal section on the right and a sagittal section on the left).

**Figure 2:**
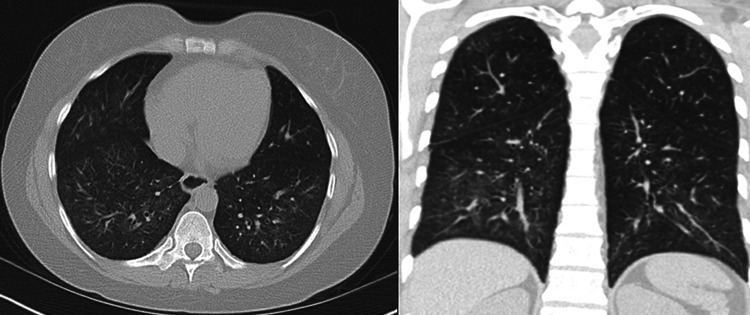
Repeat CT scan of the chest two years after she started on belimumab showing marked improvement of the bilateral lower lobes ground-glass opacity (coronal section on the right and a sagittal section on the left).

A 39-year-old female presented to our multidisciplinary pulmonary and rheumatology clinics complaining of worsening shortness of breath, skin sclerosis of the face, upper chest, and upper extremities. It was associated with pain and stiffness of elbows, wrists and fingers. rheumatological tests including ANA, anti-Smith and RNP, anti-U1 RNP, fibrillarin U3 RNP antibodies were positive. She was diagnosed with systemic lupus erythematosus (SLE) and scleroderma overlap syndrome. The initial pulmonary function test (PFT) in November 2014 showed forced vital capacity (FVC), total lung capacity (TLC) of 1.72 L (58%), and 2.74 (57%) but she could not perform the maneuvers to correctly test for the diffusion capacity of carbon monoxide (DLCO). She was only able to walk 172 meters on a 6-minute walk test and her oxygen saturation decreased from 99% to 92% while breathing room air. High-resolution computer tomogram (HRCT) of the thorax showed ground-glass opacities consistent with nonspecific interstitial pneumonitis pattern (NSIP) (Figure 1). She was started on hydroxychloroquine 400 mg twice daily and cyclophosphamide 100 mg orally daily for treatment of scleroderma/SLE overlap and ILD. An echocardiogram revealed estimated right ventricular systolic pressure of 50 mmHg concerning for pulmonary hypertension. She underwent a right heart catheterization which showed a mean pulmonary artery pressure (mPAP) of 29 mmHg, pulmonary capillary wedge pressure of 10 mmHg and cardiac output was 4 L/min. She was hence diagnosed with pulmonary arterial hypertension (PAH) secondary to scleroderma/SLE overlap and was treated with tadalafil 40 mg daily and ambrisentan 10 mg daily. After two months, she started having hematuria and was diagnosed with cyclophosphamide-induced hemorrhagic cystitis. Cyclophosphamide was discontinued and was started on azathioprine 100 mg twice daily which was later increased to 150 mg twice a day. The patient continued to complain of dyspnea. In the summer of 2016, her PFT showed worsening of lung function and she started requiring oxygen with exertion. HRCT showed persistent opacities. At that point, it was decided to add belimumab 750 mg every four weeks to her therapeutic regimen. After three doses, she started noticing some clinical improvements and she had a significant improvement on follow-up PFTs. She could now obtain DLCO which was then 4.35 ml/min/mmHg/L (Table 1). Two years later, her TLC and 6-minute walk distance improved by 550 mL and 152 meters, respectively. Ground glass opacities on follow-up HRCT scan improved (Figure 2) and right ventricular systolic pressure on echocardiogram normalized. Currently, the patient has no limitations with exercise. She maintains 100% oxygen saturation on the 6-minute walk test and walked 345 meters on the latest test. HRCT scan findings have been stable.

**Table 1:** Pulmonary function test showing the tread in the changes of the pulmonary function test indices. FVC - Forced vital capacity; FEV1 - Forced expiratory volume in 1 second; SVC - Spontaneous vital capacity; TLC - Total lung capacity; RV - Residue Volume; DLCO - diffusion capacity for carbon monoxide; 6-MWT - 6-minute walk test

	FVC	FEV1	FEV1/FVC	SVC	TLC	RV	DLCO	6-MWT
Test Date	L	L	%	L	L	L	mL/min/mmHg	Meters (m)
November 2014	1.72	1.72	100	1.05	2.74	1.69		172
August 2015	1.42	1.42	100	1.13	2.28	1.15		
October 2016	1.99	1.99	100	1.84	3.02	1.18	4.35	191
March 2017	2.14	1.9	89	1.81	2.92	1.11		235
August 2018	2.36	2.07	88	2.22	3.57	1.35	12.28	384

## Discussion

CTDs are frequently associated with ILD with considerable morbidity and mortality. The ILDs represent a group of heterogeneous parenchymal lung disorders characterized by inflammation with or without fibrosis and presents with diverse clinical and radiological patterns [1]. Systemic sclerosis, rheumatoid arthritis, SLE, mixed CTD, Sjogren’s disease, and inflammatory myopathies including polymyositis, dermatomyositis, and anti-synthetase syndrome are the most common connective tissue disorders associated with ILD [1]. CTD-associated ILD (CTD-ILD) can present as NSIP, usual interstitial pneumonitis (UIP), organizing pneumonia, rarely lymphocytic interstitial pneumonitis (LIP), or in combinations of either of those [1]. Our patient was diagnosed with NSIP based on the clinical and HRCT scan findings. Despite recent advances in the understanding of the pathogenesis of fibrosing lung disorders, optimal management of CTD-ILD has remained a big challenge largely due to significant disease heterogeneity and the lack of large randomized controlled trials to guide treatment. The approach to treatment, therefore, requires multidisciplinary collaboration between the rheumatologist, pulmonologist, chest radiologist, and/or pathologist [1]. Our patient was started on an aggressive regimen with cyclophosphamide and hydroxychloroquine due to her presentation with ILD in addition to pulmonary vasodilators and pulmonary hypertension.

Conventional disease-modifying anti-inflammatory drugs (DMARDs) are effective in the treatment of CTD but their efficacy in treating CTD-ILD is not clear. Corticosteroids, which have anti-inflammatory and immunosuppressive properties, are used as the initial therapy of CTD-ILD to stabilize the disease [2]. However, their long-term use is avoided due to significant side effects and the absence of data supporting their efficacy [2]. Cyclophosphamide, an alkylating agent with potent immunosuppressant and immunomodulation effects has been shown to be efficacious in stabilizing and in some instances improving lung function in systemic sclerosis-associated ILD [3]. During the scleroderma lung study (SLS 1), cyclophosphamide demonstrated improvement in FVC and dyspnea and quality of life [3]. However, it is associated with a high frequency of adverse events including life-threatening hemorrhagic cystitis like in our patient and the benefits were lost at six months of stopping treatment [3]. Mycophenolate Mofetil (MMF) is a pro-drug of mycophenolic acid, with a potent cytostatic effect on lymphocytes function. Various studies including scleroderma lung study II (SLS II) have demonstrated that MMF, used in treatment maintenance, improves, or stabilizes lung function and is well tolerated [4,5]. Other conventional DMARDs, azathioprine, and tacrolimus have also demonstrated efficacy in select cases like CTD-ILD due to inflammatory myositis [6,7].

In a recent study, anti-fibrotic medications such as nintedanib and pirfenidone have shown efficacy in reducing the decline of the FVC in patients with progressive pulmonary fibrosis ILD such as seen in subset scleroderma and rheumatoid arthritis [8]. With the advances in understanding the biological and disease mechanisms, some have advocated combining antifibrotics with immunosuppressive medications. However, this approach is limited by adverse events and a high rate of therapy discontinuation. Hence, search for optimal therapy, including novel agents is needed [1]. Their use is not applicable in those with inflammatory disease such as non-specific interstitial pneumonitis or acute interstitial pneumonitis.

Rituximab, a biologic DMARD have been evaluated in the treatment of CTD-ILD and is shown to stabilize lung function and imaging in multiple observational and retrospective studies [9-11]. Rituximab is well tolerated with side effects profile comparable to other DMARDs though there have been reports of acute or subacute exacerbation of ILD [11]. The efficacy of other biologics including ticilimumab and abatacept in the treatment of CTD-ILD is unknown.

Our patient responded to belimumab, a fully human IgG1λ recombinant monoclonal antibody directed against BLyS (B lymphocyte stimulator). Specific binding of belimumab with the soluble BLyS prevents the interaction of BLys with its receptors and indirectly decreases the B-cell survival and production of autoantibodies [12]. As in the other studies [12-14], additional belimumab to other immunosuppressant agents in our patient resulted in clinical and radiological improvement. This suggests that this novel disease-modifying drug, when combined with standard of care, has the potential to substantially delay progression and/or improve CTD-ILD outcomes including improved exercise capacity and lung functions.

## Conclusions

In conclusion, auto-immune diseases are associated with CTD-ILD with significant morbidity and mortality. It is therefore critical to evaluate for ILD in patients with unexplained dyspnea and hypoxia. In most cases, CTD-ILD responds to immunosuppressive therapy directed to the auto-immune conditions, however in some cases treatment directed specifically to ILD may be necessary. Unfortunately, therapeutic options for CTD-ILD are limited. According to our case report, the additional belimumab resulted in sustained clinical and radiological improvements. To our knowledge, there has been no prior study to quantify the effects of Belimumab or other biologics on CTD-ILD. More targeted therapies are needed for this devastating disease.
